# Effects of gender and age on sleep EEG functional connectivity differences in subjects with mild difficulty falling asleep

**DOI:** 10.3389/fpsyt.2024.1433316

**Published:** 2024-07-09

**Authors:** Xiaodong Luo, Bin Zhou, Jilong Shi, Gang Li, Yixia Zhu

**Affiliations:** ^1^ Psychiatry Department, The Second Hospital of Jinhua, Jinhua, China; ^2^ College of Mathematical Medicine, Zhejiang Normal University, Jinhua, China; ^3^ College of Engineering, Zhejiang Normal University, Jinhua, China

**Keywords:** sleep, sleep stages, electroencephalography (EEG), polysomnography (PSG), machine learning, functional connectivity

## Abstract

**Introduction:**

Difficulty falling asleep place an increasing burden on society. EEG-based sleep staging is fundamental to the diagnosis of sleep disorder, and the selection of features for each sleep stage is a key step in the sleep analysis. However, the differences of sleep EEG features in gender and age are not clear enough.

**Methods:**

This study aimed to investigate the effects of age and gender on sleep EEG functional connectivity through statistical analysis of brain functional connectivity and machine learning validation. The two-overnight sleep EEG data of 78 subjects with mild difficulty falling asleep were categorized into five sleep stages using markers and segments from the "sleep-EDF" public database. First, the 78 subjects were finely grouped, and the mutual information of the six sleep EEG rhythms of δ, θ, α, β, spindle, and sawtooth wave was extracted as a functional connectivity measure. Then, one-way analysis of variance (ANOVA) was used to extract significant differences in functional connectivity of sleep rhythm waves across sleep stages with respect to age and gender. Finally, machine learning algorithms were used to investigate the effects of fine grouping of age and gender on sleep staging.

**Results and discussion:**

The results showed that: (1) The functional connectivity of each sleep rhythm wave differed significantly across sleep stages, with delta and beta functional connectivity differing significantly across sleep stages. (2) Significant differences in functional connections among young and middle-aged groups, and among young and elderly groups, but no significant difference between middle-aged and elderly groups. (3) Female functional connectivity strength is generally higher than male at the high-frequency band of EEG, but no significant difference in the low-frequency. (4) Finer group divisions based on gender and age can indeed improve the accuracy of sleep staging, with an increase of about 3.58% by using the random forest algorithm. Our results further reveal the electrophysiological neural mechanisms of each sleep stage, and find that sleep functional connectivity differs significantly in both gender and age, providing valuable theoretical guidance for the establishment of automated sleep stage models.

## 1 Introduction

Sleep occupies one-third of human life (1). The daily alternation between sleep and awakening is one of the most important characteristics of our life (2). The functions of sleep are diverse (3). Looking back on the research in the past ten years, we find that sleep can enhance immune defense ability (4), improve cognition, and promote memory consolidation (5, 6). In the short term, lack of sleep can lead to impaired memory and attention (7). In the long run, it will lead to neurological disorders and even death (8–10). More and more evidence shows that sleep disorder can lead to cognitive decline (11), and increase the risk of Alzheimer’s disease (12). In addition, sleep deprivation is positively correlated with a higher risk of cardiovascular disease (13) and higher all-cause mortality (14). Insomnia is a predictor of depression and anxiety (15), and it is also associated with suicidal thoughts and behaviors (16). According to epidemiological statistics, the prevalence rate of insomnia is about 10-20%, of which about 50% develop a chronic course (17). Among them, children are mainly affected by sleep bruxism, temporomandibular joint disorder (TMD) and untreated dental caries negatively producing insomnia (18). TMD also severely interferes with the quality of sleep in adults, leading to a concomitant decrease in life satisfaction. Moreover, as a result of transcranial magnetic stimulation effects, women demonstrated significantly worse sleep quality as compared to men (19). Insomnia is also particularly common in the elderly, with more than 50% suffering from insomnia due to disease or aging factors (20). Therefore, sleep disorders influence the quality and satisfaction of life in general, not only influencing a lot of factors. Sleep diseases have caused a very heavy burden on the medical system, social economy, and human life (21, 22). The related research on sleep is very urgent.

Sleep disorders may be caused by physiological factors such as gender, age, eating disorders, mental factors such as adverse psychological and physiological reactions, environmental factors such as changes in light and sound intensity, lifestyle and behavioral factors such as addiction to coffee, alcohol, and cigarettes, as well as illnesses such as depression and anxiety. It usually shows abnormalities such as sleepwalking, night terrors, nightmares, snoring, teeth grinding, excessive sweating, sleep talking, and easy waking. Therefore, sleep can be analyzed from a number of parameters such as electroencephalogram (EEG), ophthalmoplegia, mandibular electromyography, oro-nasal airflow and respiratory motility, cardioplegia, oximetry, snoring, limb movements, body position, and so on. Polysomnography (PSG) can record many of these biosignals throughout the sleep process (23), and is the most commonly used technique for sleep evaluation and disease diagnosis in medicine, and is becoming more and more popular both at home and abroad.

Sleep staging is an important process of analyzing PSG data, an important part of sleep assessment, and the basis of disease diagnosis (24–26). The brain activity in each stage of sleep is not in a static state, but a series of periodic changes of active regulation. In order to define the sleep process in a unified standard, the “Interpretation Manual of Sleep and Related Events” issued by the American Academy of Sleep Medicine (AASM) divides sleep into five different stages: awake (W) stage, non-rapid eye movement (NREM: N1, N2, N3) stage, and rapid eye movement (REM) stage (27). Sleep staging is often applied in a variety of ways, including the assessment of sleep quality (28) (such as sleep duration, sleep depth, and sleep efficiency), the investigation of neural mechanisms (29), and the evaluation of medication efficacy on sleep (30). For example, REM sleep abnormalities have been associated with neurodegenerative diseases, and are therefore often utilized in clinical studies for diagnostic studies and efficacy assessment of diseases such as Parkinson’s disease (31) and Alzheimer’s disease (32). Sleep stages are usually interpreted by sleep experts (27). Manual interpretation is time-consuming and labor-intensive, vulnerable to subjective influence, unsuitable for processing large-scale data, and unable to meet the needs of millions of patients with sleep disorders (33). In order to improve efficiency and reduce labor costs, researchers tried to use sleep EEG (from PSG) and artificial intelligence to construct an automatic sleep staging method (34, 35).

Sleep EEG features are commonly used to explore sleep staging (36). According to research reports, significant differences in EEG features in different sleep stages, such as power spectrum features, nonlinear dynamics features, and functional connections (37–39). Cantero et al. pointed out that alpha wave power is an important feature of human REM sleep (40). Miskovic et al. found that in the whole sleep cycle, the change of entropy strongly depends on the time scale, and slow-wave sleep is characterized by the decrease of entropy in the short time scale and the increase of entropy in the long time scale (41). In a previous study exploring the temporal evolution of the power spectrum and coherence spectrum between cerebral hemispheres, the power spectrum and coherence spectrum showed obvious peaks in the NREM stage, but did not in REM sleep (42). In a study of sleep staging based on a single-channel EEG signal using 22 features in the time domain, time-frequency, and nonlinear analysis methods, the highest accuracy of sleep staging is 85.93% by comparing sample entropy, fuzzy entropy, fractal dimension and complexity as feature parameters (43). In another study, 18 features extracted from EEG, ECG, and EMG in the time domain and frequency domain were extracted to construct feature vectors, and the accuracy of sleep staging was 82.53% (44). The existing researches on sleep staging are mainly based on time-domain, frequency-domain, and nonlinear EEG features of a single or a few channels. These features can only obtain local features, while ignoring the global information between different brain regions.

Brain functional connections are considered to be closely related to brain activity, which can be used to study the interaction between brain regions (45). Functional connectivity is a statistical concept, that can quantify the time dependence of neuron activation patterns in morphologically and physiologically different brain regions by using statistical methods such as Pearson correlation coefficient, spectral coherence estimation, phase-locked value, and mutual information (MI) (46, 47). Functional connectivity is widely used in disease research, such as exploring neuroregulatory factors in depression treatment (48), predicting epileptic seizures, and locating epileptogenic foci (49). The combined use of EEG and functional connectivity may be a powerful tool to study the basis of subconscious function under physiological and pathological conditions (50). Functional connections are quite different in different sleep stages, so they can be used for sleep staging. In a study of analyzing brain interaction in different sleep stages by functional connectivity analysis method, it is found that the functional connectivity strength increases in low-frequency bands (delta and alpha bands) at different stages of NREM, and the classification accuracy is high (51). To sum up, it is suggested that functional connections may be an effective feature to distinguish different stages of sleep. In this study, mutual information is used to determine the functional connectivity due to its ability to comprehensively evaluate the amplitude and phase information between EEG channels, which is then applied for exploring the changes and laws of functional connections in different sleep stages.

In existing studies of sleep staging based on EEG features, it has been shown that the accuracy of automated sleep staging based on different EEG features is generally not high. It may be related to not considering physiological factors such as age and gender, and also the effects of gender and age on the connectivity of sleep EEG function were not clearly revealed. The brain connectivity research report points out that the functional network will change with age (52). Large-scale data research found that Japanese sleep characteristics (time, duration, and quality) are significantly different from age and gender (53). In the study of insomnia patients, women sleep longer than men, the relative beta power of women is higher than that of men, the dominant frequency of the occipital lobe of elderly patients is slower than that of young patients, and the age effect of women is more obvious in clinical variables and quantitative EEG (54). In order to improve the accuracy of sleep staging, it is necessary to analyze the influence of gender and age on sleep EEG function.

To sum up, this study will explore age and gender differences in sleep EEG functional connectivity. A finer division of gender and age groups will be made, and the research work will be carried out in two dimensions: statistical analysis and machine learning validation, with the aim of providing valuable theoretical guidance for automated sleep staging models.

## 2 Materials and methods

### 2.1 Data introduction

The data for this study were downloaded from the Sleep-EDF database (Sleep-EDF Database Expanded v1.0.0 (physionet.org)). Sleep-EDF database has two datasets, one of which is a study of the effect of age on sleep in subjects that had mild difficulty falling asleep but were otherwise healthy. For this study, two full nights of sleep data from 78 subjects with a mean age of 56.01 ± 22.20 (36 females, and 42 males, aged 25-101 years) in the SC dataset were selected, with a total of 156 samples, and due to incomplete data in 21 of them, a total of 135 samples were finally included in the analysis. As shown in **Table 1**, there were a total of 39 samples of young subjects aged 25-45 years, 56 samples of middle-aged subjects aged 46-69 years, and 40 samples of elderly subjects aged 70-101 years. As shown in **Table 2**, all samples consisted of 56 samples of male subjects with a mean age of 57.89 ± 21.34, and 79 samples of female subjects with a mean age of 54.39 ± 21.65. There was no statistically significant difference in the mean age of the male and female groups. All subjects were not taking sleep-related medications. Sleep EEG signals from bipolar leads Fpz-CZ and PZ-OZ with a sampling frequency of 100 Hz were recorded for each subject.

**Table 1:** Statistical results of samples divided by age.

	25-45: Young	46-69: Middle	70-101: Old
Average age	28.69 ± 2.93	57.95 ± 6.61	81.375 ± 10.04
Sample size (male/female)	19/20	19/37	18/22

**Table 2:** Statistical results of samples divided by gender.

	Female	Male
Number of samples	56	79
Average age	57.89 ± 21.34	54.39 ± 21.65

### 2.2 EEG signal preprocessing

Sleep experts have manually labeled the sleep data of each sample into eight categories according to R&K standards. Combined with the AASM standard formulated by the American Academy of Sleep Medicine, the N3 and N4 stages are combined into an N3 stage, and motion and unknown data are removed. Therefore, the sleep stages of each sample are divided into five categories, W, N1, N2, N3, and REM. In this study, the 30s sleep EEG signals are classified as a segment of data, and 0.5-30Hz band-pass filtering is carried out by a Butterworth filter. Then six rhythm waves are extracted from two leads of EEG signals, and the features of each rhythm wave are extracted on this basis. See **Table 3** for the division of six rhythm frequency bands.

**Table 3:** The six rhythm band division schemes used in this study.

Rhythm	Frequency range
Delta (δ)	0.5-4Hz
Theta (θ)	4-8Hz
Alpha (α)	8-13Hz
Beta (β)	13-30Hz
Sleep spindle	11-16Hz
Sawtooth wave	3-7Hz

### 2.3 Functional connectivity calculation

Functional connectivity is a brain science analysis method used to study the interaction between different brain regions. By measuring the signals between different regions of the brain, the interaction between these regions in different tasks or states is studied. In the functional connectivity representation method, Mutual Information (MI) can measure the interaction degree between different brain regions from both amplitude and phase shown in **Equation 1**. Let the joint distribution of two random variables (*X*, *Y*) be *p*(*x*, *y*) and the marginal distribution be *p*(*x*), *p*(*y*), the mutual information *I*(*X*; *Y*) is the relative entropy of the joint distribution *p*(*x*, *y*) and the marginal distribution *p*(*x*), *p*(*y*):


(1) $$I\left(X;Y\right)=\sum_{x\in X}\sum_{y\in Y}p\left(x,y\right)\log\frac{p\left(x,y\right)}{p\left(x\right)p\left(y\right)}$$

### 2.4 Classification models and evaluation metrics

In this study, in order to corroborate whether differences in brain function among people of different genders and ages, three machine learning models, namely, Support Vector Machine (SVM), Random Forest (RF), and K-Nearest Neighbor (KNN), based on functional connectivity features, were used to perform sleep staging tests on samples of different genders and ages. The following will introduce the characteristics of the 3 algorithms respectively.

SVM is a supervised learning algorithm, which is widely used in the field of pattern recognition and data classification. Its core idea is to maximize the interval between data points of different categories by finding the optimal hyperplane, so as to achieve effective classification of data.

KNN algorithm uses the training sample dataset with known categories to classify the new samples by calculating the distance between the test new samples and the samples in the training set. The classification of the KNN algorithm is based on the category which is the majority of the *K* nearest neighbors of the new samples. In this study, the *K* of the KNN algorithm is set to 9.

RF is an integrated learning method constructed based on the decision tree, which improves the performance of classification and regression by randomly selecting features and samples, constructing multiple decision trees, and fusing their prediction results. The number of decision trees of Random Forest is set to 50 for this study.

Since this dataset has a sample imbalance problem, the sample numbers of N1 and N3 are significantly less than those of other categories, down-sampling is adopted in this experiment. In the down-sampling process, we randomly remove some samples from the categories with more samples to make the number of samples from different categories more balanced. Ten rounds of training are repeated, and in each round, the dataset is randomly divided into the training set and test set according to 7:3. Finally, the four metrics: true positives (*TP*), false negatives (*FN*), true negatives (*TN*), and false positives (*FP*) are utilized to calculate the *Accuracy* (the proportion of correctly categorized samples to the total number of samples), *Precision* (the proportion of correctly predicted positive classifications to all the samples that are predicted to be positively categorized), *Recall* (which refers to the proportion of correctly predicted positive classifications to all the samples that are actually positively categorized), and the *F*1 score (the harmonic mean of precision and recall) of different models. The specific formulas are given in **Equations 2**–**5**:


(2) $$Accuracy=\frac{TP+TN}{TP+TN+FP+FN}$$

(3) $$Precision=\frac{TP}{TP+FP}$$

(4) $$Recall=\frac{TP}{TP+FN}$$

(5) $$F1=\frac{2\times TP}{2\times TP+FP+FN}$$

### 2.5 Statistical analysis method

One-way analysis of variance (one-way ANOVA) was performed to determine the significant statistical differences in EEG functional connectivity features among the groups. First, one-way repeated-measures ANOVA was done between groups, and if statistically different, then pairwise comparisons after multiple tests were performed. Statistical differences were considered significant when *P*< 0.05. All statistical analysis was carried out using MATLAB 2019b software.

## 3 Results

**Figure 1:**
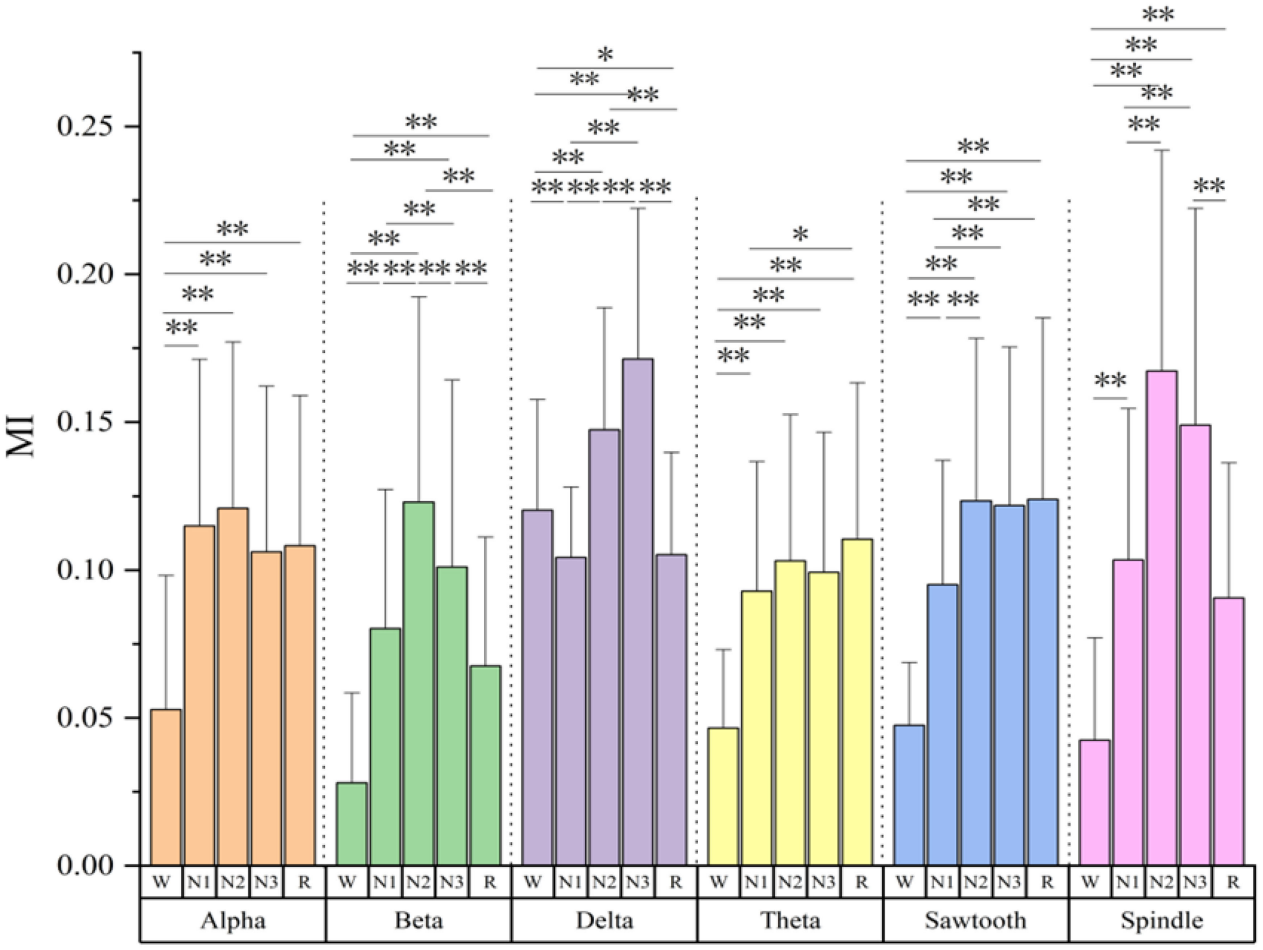
Mutual information values of six sleep rhythm waves alpha, beta, delta, theta, sawtooth, and spindle in different sleep stages of all subjects. * means *p*< 0.05, ** means *p<* 0.01.

**Figure 1** shows the MI corresponding to the six sleep rhythm waves in different sleep stages for all subjects. The results show that most of the sleep rhythm waves corresponded to MIs that were significantly different in pairwise comparisons between different sleep stages. The most significant differences in beta and delta waves mutual information were found between all sleep stages (*p*< 0.05), except for the N1 and R stages, which did not differ significantly. In addition, significant differences of MI in spindle wave were found between all stages except between stages N2 and N3, and between stages N1 and R (*p*< 0.05), where no significant differences existed. For the MI of the sawtooth wave, there were significant differences between all stages except between N2, N3, and R (*p*< 0.05). The MI of the theta wave had significant differences between the W stage and other stages (*p*< 0.05), and between the N1 and R stages (*p<* 0.05), and the rest had no significant differences. The alpha wave had the smallest difference in MI, and only the W stage had a significant difference from other stages (*p<* 0.05), and the differences between the N1, N2, N3, and R stages were all non-significant. Except for the delta wave, the mutual information values of the remaining five rhythmic waves were lower in stage W than in the other sleep stages.

**Figure 2:**
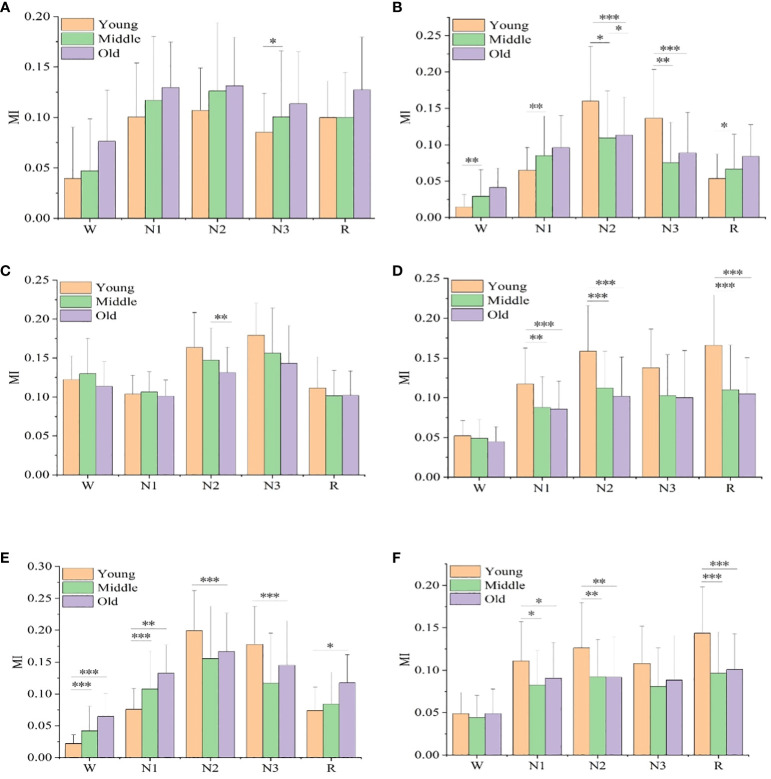
Mutual information values of six sleep rhythm waves, alpha, beta, delta, sawtooth, spindle, and theta, in different age groups at different sleep stages. **(A)** shows the MI value of alpha wave, **(B)** shows the MI value of beta wave, **(C)** shows the MI value of delta wave, **(D)** shows the MI value of sawtooth wave, Figure **(E)** shows the MI value of spindle wave, and **(F)** shows the MI value of theta wave. * means *p*< 0.05, ** means *p*< 0.01, *** means *p*< 0.001.

**Figure 2** shows the mutual information values of the six sleep rhythm waves in different age groups of subjects with different sleep stages. As shown in **Figure 2A**, the α-wave mutual information values were statistically significant only between the young and middle-aged groups at stage N3 (*p*< 0.05), while there was no statistical difference between the other stages and age groups. As shown in **Figure 2B**, the β-wave mutual information was statistically different between all age groups in stage N2 (*p*< 0.05), especially more significant in the young and elderly groups (*p*< 0.001); it was a significant difference between the young and middle-aged groups in stages W, N1, N3, and R (*p*< 0.05); and it was even a highly significant difference between the young and elderly groups in stage N3 (*p*< 0.01); however, in stages W, N1, N3, and R, both the middle-aged group and the elderly group differences were not statistically significant. As shown in **Figure 2C**, the δ-wave mutual information values were only significantly different between the middle-aged group and the old-aged group in the N2 stage (*p*< 0.01), while no statistically significant difference was found between the other stages and age groups. As shown in **Figure 2D**, for the sawtooth wave mutual information, a highly significant difference was found between the youth group with the middle-aged, and elderly groups at the N2 and R stages (*p*< 0.001), and a significant difference was found between the youth group with the middle-aged, and elderly groups at the N1 stage (*p*< 0.01), but there was no significant difference between all other stages and age groups. As shown in **Figure 2E**, in stages W and N1, the mutual information of the spindle wave was significantly different between the young group with the middle-aged, and old-aged groups (*p*< 0.01); in stages N2 and N3, the differences were highly significant between the young and the old-aged groups (*p*< 0.001); the differences between the young and the old-aged groups were statistically significant in stage R (*p*< 0.05); however, there were no significant differences in all the other stages and among the age groups. As shown in **Figure 2F**, the theta wave mutual information value has a generally significant difference between the young with middle-aged groups, and the old age group in the N1 stage (*p*< 0.05), a significant difference between the young with middle-aged groups, and the old age group in the N2 stage (*p*< 0.01), and a highly significant difference between the young with middle-aged groups, and the old age group in the R stage (*p*< 0.001); but with no significant difference between all other stages and age groups.

**Figure 3:**
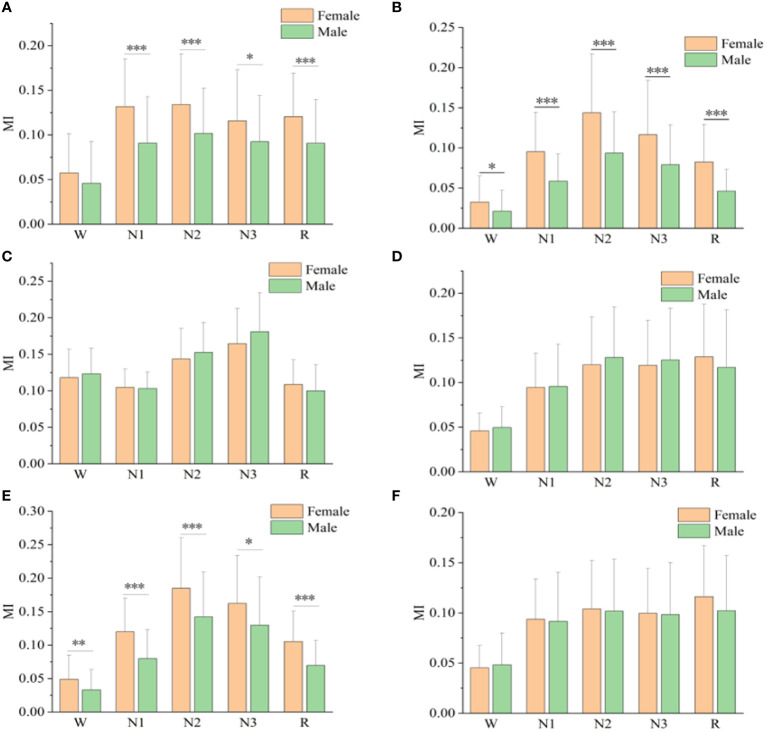
Mutual information values of alpha**(A)**, beta**(B)**, delta**(C)**, sawtooth**(D)**, spindle**(E)**, and theta**(F)** sleep rhythm waves of different genders at different sleep stages. * means *p*< 0.05, ** means *p*< 0.01, *** means *p*< 0.001.

**Figure 3** shows the mutual information values of the six sleep rhythm waves in each sleep stage for different gender groups. As shown in **Figure 3A**, the α-wave mutual information values of the remaining 4 stages, except for stage W, were statistically different between different gender groups (*p*< 0.05). As shown in **Figure 3B**, for the β-wave mutual information values, statistically significant differences existed between different gender groups for all sleep stages (*p*< 0.05), with highly significant differences for stages N1, N2, N3, and R (*p*< 0.001). As shown in **Figure 3E**, the mutual information values for the spindle wave were statistically different between the different gender groups for all sleep stages (*p*< 0.05), with highly significant differences for stages N1, N2, and R (*p*< 0.01). As shown in **Figures 3C, D, F**, the differences in δ-, sawtooth-, and θ-wave mutual information values were not statistically significant among different gender groups in each stage of sleep.

Using the functional connectivity features of all sleep rhythms as inputs, the sleep staging task was accomplished based on SVM, KNN, and RF classifiers, and the performance is shown in **Table 4**. The results show that the RF classifier has the highest average accuracy of 50.14 ± 0.02%, SVM has the second highest accuracy of 49.67 ± 0.01%, and KNN has an accuracy of 49.35 ± 0.01%. In addition, the classification accuracies of different age and gender groups (young female, young male, middle-aged female, middle-aged male, old female, old male) were also calculated separately, and it remained that the accuracy of RF was the highest in all groups, with 62.61 ± 0.02%, 58.20 ± 0.02%, 52.49 ± 0.01%, 48.41 ± 0.01%, 47.89 ± 0.01%, and 52.70 ± 0.02%. The accuracy of automated sleep staging varied considerably across age and gender groups, with the young female group having the highest accuracy, 12.47% higher than the all-sample group, whereas the middle-aged male and older female groups had slightly lower accuracy than the all-sample group. More surprisingly, the average accuracy rate of the subgroups (53.72%) was still higher than that of the full sample group (50.14%).

**Table 4:** Sleep staging classification performance results for different subgroups.

Models	Groups	Accuracy	Sensitivity	Specificity	F1
**SVM**	Young Female	60.52 ± 0.02	61.93 ± 0.01	60.64 ± 0.02	61.28 ± 0.02
	Young Male	55.37 ± 0.02	55.99 ± 0.02	56.39 ± 0.02	56.18 ± 0.02
	Middle-aged Female	49.16 ± 0.02	50.09 ± 0.01	49.82 ± 0.02	49.95 ± 0.01
	Middle-aged Male	42.87 ± 0.02	42.44 ± 0.02	48.10 ± 0.03	45.09 ± 0.02
	Elderly Female	44.07 ± 0.01	45.05 ± 0.02	48.37 ± 0.03	46.65 ± 0.02
	Elderly Male	47.36 ± 0.01	48.36 ± 0.02	47.94 ± 0.01	48.15 ± 0.01
	All	49.67 ± 0.01	50.45 ± 0.01	51.20 ± 0.02	50.82 ± 0.02
**KNN**	Young Female	60.81 ± 0.01	61.32 ± 0.01	60.69 ± 0.02	61.00 ± 0.01
	Young Male	57.88 ± 0.00	57.63 ± 0.00	58.53 ± 0.01	58.08 ± 0.00
	Middle-aged Female	51.02 ± 0.01	51.00 ± 0.02	51.30 ± 0.01	51.15 ± 0.01
	Middle-aged Male	46.99 ± 0.02	46.90 ± 0.02	47.47 ± 0.02	47.18 ± 0.02
	Elderly Female	47.14 ± 0.01	47.40 ± 0.02	47.322 ± 0.01	47.36 ± 0.01
	Elderly Male	51.75 ± 0.02	52.18 ± 0.02	51.76 ± 0.02	51.97 ± 0.02
	All	49.35 ± 0.01	49.37 ± 0.01	49.73 ± 0.02	49.55 ± 0.01
**RF**	Young Female	62.61 ± 0.02	62.97 ± 0.02	62.50 ± 0.02	62.73 ± 0.02
	Young Male	58.20 ± 0.02	58.06 ± 0.02	58.60 ± 0.02	58.33 ± 0.02
	Middle-aged Female	52.49 ± 0.01	52.02 ± 0.02	53.21 ± 0.01	52.60 ± 0.01
	Middle-aged Male	48.41 ± 0.01	47.94 ± 0.01	49.21 ± 0.01	48.56 ± 0.01
	Elderly Female	47.89 ± 0.01	47.85 ± 0.02	48.16 ± 0.01	48.00 ± 0.01
	Elderly Male	52.70 ± 0.02	52.57 ± 0.02	53.14 ± 0.02	52.85 ± 0.02
	All	50.14 ± 0.02	49.83 ± 0.02	50.69 ± 0.02	50.26 ± 0.02

## 4 Discussion

This study aims to explore the differences of age and gender in sleep electrical brain connectivity based on statistical analysis and machine learning validation. The main findings are as follows: First, the functional connections of each sleep rhythm wave have great differences in different sleep stages, the functional connections of delta and beta can significantly distinguish each sleep stage. Second, there were significant differences in functional connectivity between young people with middle-aged, and young people with old people in different age groups, while there were no significant differences between middle-aged and old people. Third, in different genders, the functional connectivity strength of female high-band is generally higher than that of male, while the difference in low-band is not significant. The results of these analyses are discussed in more detail below.

### 4.1 Functional connectivity features of effective sleep rhythms for sleep staging

In this study, mutual information is used as a measure of functional connectivity to study the changes in functional connectivity of each sleep rhythm wave in different sleep stages. The results show that the functional connectivity of each sleep rhythm wave has great differences in different sleep stages. What is most worth mentioning is that beta and delta have the most significant differences in functional connectivity in each sleep stage. With the deepening of NREM sleep, the mutual information values of beta and delta increased, suggesting coupling between the two EEG signals, increased dependence between cortical regions, and increased sharing of information. This finding is similar to the findings of a recent study that explored the commonality of EEG functionally connected populations, with functional connectivity intensity increasing significantly in the delta and beta bands as NREM sleep deepened (55). In addition to mutual information as a functional connectivity metric, other feature extraction methods for sleep EEG, such as the power method, also have strong specificity in beta and delta bands. In a study that used 73 features in the time domain to classify sleep stages, the EEG power ratio of delta and beta was the most successful measure for distinguishing between awake and deep sleep (classification error of 1%). It was also the single best performing measure in distinguishing all five stages (56). In another large-scale EEG spectrum analysis, it is also verified that the mean power density of beta and delta bands is significantly different (57). Similarly, a study that analyzed changes in brain activity during sleep using a combination of signal power and two functional connectivity indicators also found that changes in delta power and connectivity were among the most relevant classification features (58). In addition, we also found that the functional connectivity strength of each sleep rhythm wave in the W stage was lower, which was considered to be related to the more active brain activity in the waking stage (59). The functional connectivity strength of each sleep wave in the N2 and N3 stages is relatively high, indicating that during deep sleep, the complexity of brain activity is reduced, which is related to the increase of shared information in the regions (60). Moreover, due to the further decline in consciousness level, the interference from external information is reduced, and the cerebral cortex interdependence and synchronization are enhanced (61).

Another interesting finding is that except for theta and sawtooth, the functional connections of the other four frequency bands are not significantly different in the N1 and REM stages. In terms of the waveform, the background wave in the REM stages is similar to the N1 waveform in EEG, which is a low amplitude wave of 3-7 Hz (62). Moreover, a study on the relationship between finger twitch and key sleep parameters suggests that the finger twitch density in N1 and REM stages is similar and difficult to distinguish (63). Because of the feature similarity between N1 and REM stages, in order to improve the accuracy of sleep staging, many studies combine stages N1 and REM into one stage to train classifiers (64, 65). However, it is still necessary to distinguish each stage clinically, and the accuracy of classifying the N1 stage is usually lower than other stages (62), so it is particularly challenging to distinguish the N1 stage from the REM stage. Therefore, it is still necessary to further find the characteristics to effectively distinguish the N1 stage from the REM stage.

### 4.2 Functional connectivity features of effective age grouping for sleep staging

According to the statistical analysis of different age groups, it is found that there are significant differences in brain functional connections between young people with old people and young people with middle-aged people, but there is no significant difference between middle-aged people and old people. For example, the functional connectivity comparison of beta, sawtooth, spindle, and theta in different age groups in different sleep stages shows that differences are significant between youth and middle age, youth and old age, especially spindle wave (sigma frequency), which is significantly different between youth and old age in each sleep stage. This is consistent with the results of another study (66), suggesting that the density, amplitude, and power of sleep spindles in the elderly are low. A study using cross-spectral coherence to evaluate the functional connectivity of EEG shows that compared with young people, the elderly show lower functional connectivity in the N2 stage, but higher connectivity in the REM and N3 stage. REM has lower EEG functional connectivity than N3, especially in young people (67). Another research combining EEG and fMRI to study brain functional connectivity found (68), that compared with young people, the connectivity between thalamus/basal ganglia and several brain regions and frontal lobe regions of various networks in the elderly decreased less, which led to slow response, increased mild sleep and piecemeal sleep, and had an age effect on sleep-dependent brain plasticity. All the above conclusions reflect that the brain functional connections were significantly different between young and old people, which is similar to our research findings. One point that cannot be ignored in our research is that no significant difference in mutual information values of all frequency bands between middle-aged and old-aged waves at different sleep stages. To sum up, the results of this study reflect that the sleep function connection of young people is quite different from that of middle-aged and old people, but no obvious difference between middle-aged and old people. The accuracy of existing sleep staging algorithms based on EEG functional connectivity is generally low, which may be related to the failure to distinguish the age of the extracted samples. According to the results of this study, age can be used as a variable, and the samples can be divided into young groups and middle old aged groups for feature extraction and modeling, in order to improve the accuracy of sleep staging.

### 4.3 Functional connectivity features of effective gender grouping for sleep staging

The group division and statistical analysis according to gender found that the EEG mutual information value of female samples in all sleep stages was generally higher than that of males, the functional connectivity difference between different genders was significant, and the strength of brain functional connectivity of female was greater than that of male. This is mainly reflected in the EEG samples in the frequency band above 8 Hz, and there is a significant difference between males and females in terms of brain functional connectivity in the high-frequency band of EEG, but no significant difference in the low-frequency. There have been many previous exploratory studies using gender as a variable, for example, in a study in which the weighted phase lag index of sleep EEG reflected the functional brain connectivity of people of different genders, the results responded to significantly greater connectivity in female than in male in the high sigma frequency range, but the opposite pattern was observed in the alpha, low sigma, and beta frequency ranges (69). Another study using the same index calculation noted that synchronization strength showed significant gender differences in all stages and bands, being higher in females during the NREM stage and higher in males during the W and REM stages in the alpha and beta bands (70). All of these findings suggest differences in functional connectivity for sleep staging by gender. This is similar to the results of the present study. In addition, we also found that the spindle has the most significant functional connectivity differences by gender, and its MI values in sleep stages were significantly different between male and female comparisons. The above findings are in perfect agreement with those in a previous study exploring gender differences in sleep neurophysiology in adolescents using sleep EEG (71), where the conclusions exposed that females have higher sleep spindle waves compared to males, which may imply stronger thalamocortical circuits in adolescent females compared to males, as well as in contrast to the low-frequency bands (< 11 Hz) of the absolute power of the sleep EEG between males and females which did not show differences. Not only in adolescents, but also in adult sleep EEG studies (69), similar gender differences exist in the alpha and sigma bands of the NREM stage, suggesting that these gender differences are generalized across the human lifespan. Moreover, it has been observed in MRI studies that both anatomical connectivity (72) and functional connectivity (73) are stronger in females. In summary, it is necessary to extract the EEG features of different genders separately as classification and recognition to improve the accuracy.

### 4.4 Analysis of sleep staging classification performance

Groups were divided for sleep staging classification according to gender and age using SVM, KNN, and RF models. The accuracy of the sleep staging results using the RF algorithm was the highest, the average accuracy of the test set overall was 50.14%, and the average accuracy of the grouping increased to 53.72%, of which the accuracy of the grouping of young women was the highest of 62.61%. Although the sleep staging performance was not so good, which may be attributed to the fact that our EEG had only two bipolar channels and a total of six functional connectivity features were extracted, there were differences in the classification accuracy between the groups, and the accuracy of the majority of the grouping was greater than the overall average, which suggests that the accuracy of the sleep staging was affected by gender and age, and grouping improved the accuracy of sleep staging. The effect of the age variable on classification accuracy has been mentioned in a few experiments on sleep staging, such as a study on sleep staging using wavelets for feature extraction and the Random Forest method for classification, the results of which found that the age of the subjects affects the performance of the model, but there is a lack of in-depth research on this (74). Even fewer studies have been conducted on sleep staging models differentiating on gender. In a previous EEG study exploring differences in brain function between adults and older adults, the SVM classifier was used with 93% accuracy when categorizing the brain by age group (52). Another study found gender differences in EEG functional connectivity between sleep stages and resting wake states based on weighted phase lag indices (70). This coincides with our findings.

### 4.5 Limitations

In this study, mutual information was used to explore age and gender differences in sleep EEG functional connectivity in individuals with mild difficulty falling asleep. Although the results of this study may be novel and valuable, some limitations should be considered. First, the current study included only 135 samples, and the results obtained can be used as a research reference and cannot provide definitive conclusions. Second, the results of sleep staging performance, although improved after fine grouping by age and gender, are not sufficient for use in practical applications, possibly due to the use of only six functionally connected features. In the future, we will further expand the sample size, analyze other EEG features and select the optimal sleep EEG features, and explore deep learning algorithms to further determine whether grouping by age and gender still improves sleep staging accuracy.

## 5 Conclusion

This study attempts to reveal the differences of age and gender in sleep EEG functional connectivity based on functional connectivity statistical analysis and machine learning validation. The findings suggest that delta and beta functional connectivity can be used as a potential electrophysiological marker for sleep staging. There were significant differences in sleep functional connectivity in both gender and age, as corroborated by the accuracy of grouping by age and gender in a sleep staging experiment based on brain functional connectivity. Therefore, in the study of, group division needs to be more carefully formulated after analyzing the changing pattern of sleep stages based on functional connectivity. The present study will provide a theoretical basis for the use of EEG functional connectivity in sleep auto staging at a deeper level.

## Data availability statement

Publicly available datasets were analyzed in this study. This data can be found here: https://physionet.org/content/sleep-edfx/1.0.0/. Further inquiries can be directed to the corresponding author(s).

## Ethics statement

Ethical approval was not required for this study of human participants in accordance with the local legislation and institutional requirements. Written informed consent from the participants was not required to participate in this study in accordance with the national legislation and the institutional requirements.

## Author contributions

XL: Writing – original draft, Writing – review & editing. BZ: Writing – original draft. JS: Writing – original draft. YZ: Writing – original draft. GL: Writing – original draft, Writing – review & editing.
